# Autologous sapheno-saphenous bypass collateral development in the setting of chronic unilateral iliac vein occlusion

**DOI:** 10.1186/s42155-018-0034-0

**Published:** 2018-10-29

**Authors:** Julie Kelman, Nicholas Xiao, Jeremy D. Collins, Jennifer K. Karp, Heron Rodriguez, Kush R. Desai

**Affiliations:** 1 Department of Radiology, Section of Interventional Radiology, Northwestern University, 676 N. St. Clair, Suite 800, Chicago, IL 60611 USA; 2 Department of Surgery, Section of Vascular Surgery, Northwestern University, Chicago, IL USA

**Keywords:** Chronic iliac vein occlusion, May-Thurner syndrome, Saphenous collaterals

## Abstract

**Background:**

Chronic iliac vein occlusion can result in the development of a variety of collateral venous drainage pathways. While several drainage pathways have been well documented, autologous sapheno-saphenous bypass collateral drainage has not been described. This novel collateral drainage pathway is readily visible on cross sectional imaging, may serve as a diagnostic indicator of chronic obstructive venous pathology, and may hint at the underlying etiology.

**Case presentation:**

This brief report depicts findings and technical considerations in two cases of venous recanalization of sapheno-saphenous collaterals in the setting of chronic unilateral iliac vein occlusion. In both cases at one-month follow-up, the patients’ pain had resolved, edema had improved, and computed tomographic venography demonstrated stent patency.

**Conclusions:**

Identification of a sapheno-saphenous collateral can provide an important clue to the underlying venous obstructive pathology, therefore guiding corrective intervention.

## Introduction

Chronic iliac vein occlusion commonly results in the development of collateral venous drainage pathways; well-described pathways include retroperitoneal, lumbar, and cross-pelvic internal iliac collaterals. However, few reports of an autologous sapheno-saphenous drainage pathway, serving as a veno-venous bypass, have been reported. This brief report describes two cases of sapheno-saphenous collaterals first noted on cross sectional imaging and subsequently confirmed venographically during successful percutaneous venous recanalization of the underlying venous occlusion.

## Case reports

A 50-year-old male with prior history of deep venous thrombosis (DVT) presented with bilateral lower extremity edema and pain. Physical examination demonstrated lipodermatosclerosis, edema, and varicose veins, affecting the left lower extremity to a significantly greater degree than the right. While on conservative management with aspirin, the patient’s Venous Clinical Severity Score (VCSS) was 9, providing indication for intervention.

Prior to the procedure, duplex examination showed evidence of non-occlusive chronic DVT in the left popliteal vein. Reflux of the great saphenous veins and small saphenous veins was seen bilaterally. Evidence of a network of bulging varix, numerous intramuscular varix, and incompetent perforators were seen bilaterally. Deep reflux was seen in the left common and femoral veins and the popliteal veins bilaterally.

**Fig. 1:**
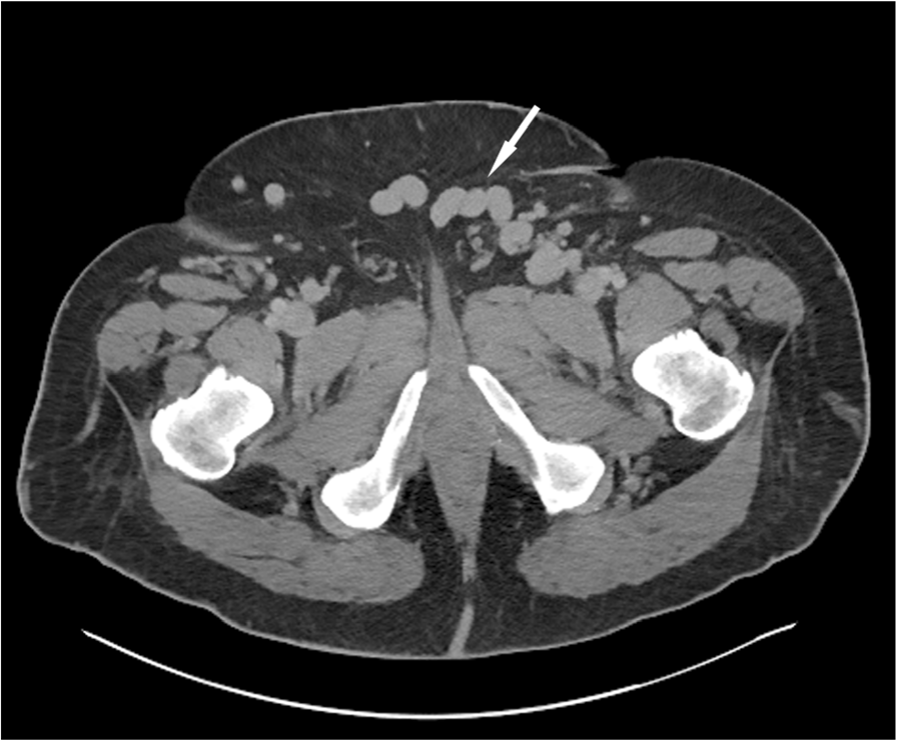
Axial CT of a 50-year old male presenting with bilateral lower extremity edema and pain. Torturous collaterals connecting the great saphenous veins immediately proximal to the saphenofemoral junctions are noted coursing through the anterior pelvic wall adipose tissue (white arrow)

Computed tomographic (CT) venography of the abdomen/pelvis demonstrated a markedly tortuous collateral vein coursing through anterior pelvic wall fat, connecting the great saphenous veins immediately proximal to the saphenofemoral junctions (Fig. 1). The left common and external iliac veins were diminutive, and there was compensatory enlargement of the right external and common iliac veins.

**Fig. 2:**
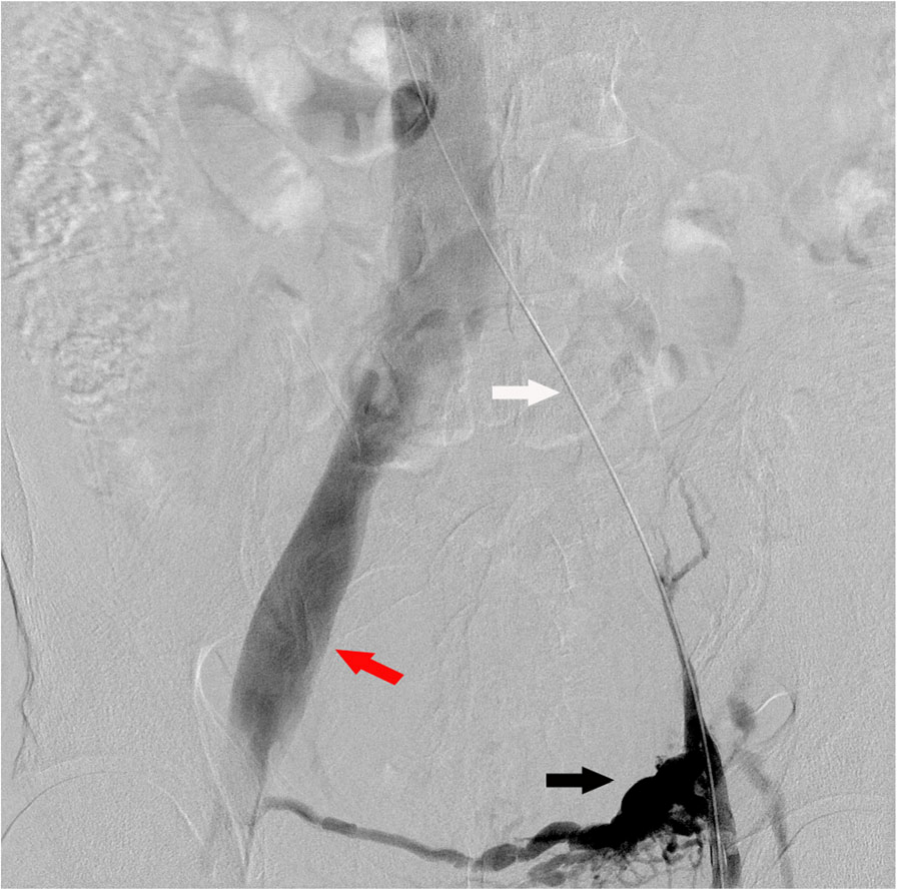
Digital subtraction venography demonstrating complete occlusion of the left common iliac vein and collateralization. The site of occlusion is identified (white arrow). Contrast injected ipsilateral and distal to the lesions is seen draining to the contralateral common iliac vein (red arrow) through the dominant left-to-right sapheno-saphenous collateral drainage pathway (black arrow)

Left greater saphenous venous access was obtained immediately proximal to the saphenofemoral junction under sonographic guidance. Venography demonstrated occlusion of the left common and external iliac veins with dominant drainage via a left to right sapheno-saphenous collateral (Fig. 2).

An 8Fr sheath was placed (Brite Tip, Cordis, Fremont, CA) and the occlusion was crossed with a stiff hydrophilic wire (Glidewire, Terumo, Tokyo, Japan) and angled 5Fr catheter (Kumpe, Cook Medical, Bloomington, IN) to obtain access into the inferior vena cava.

**Fig. 3:**
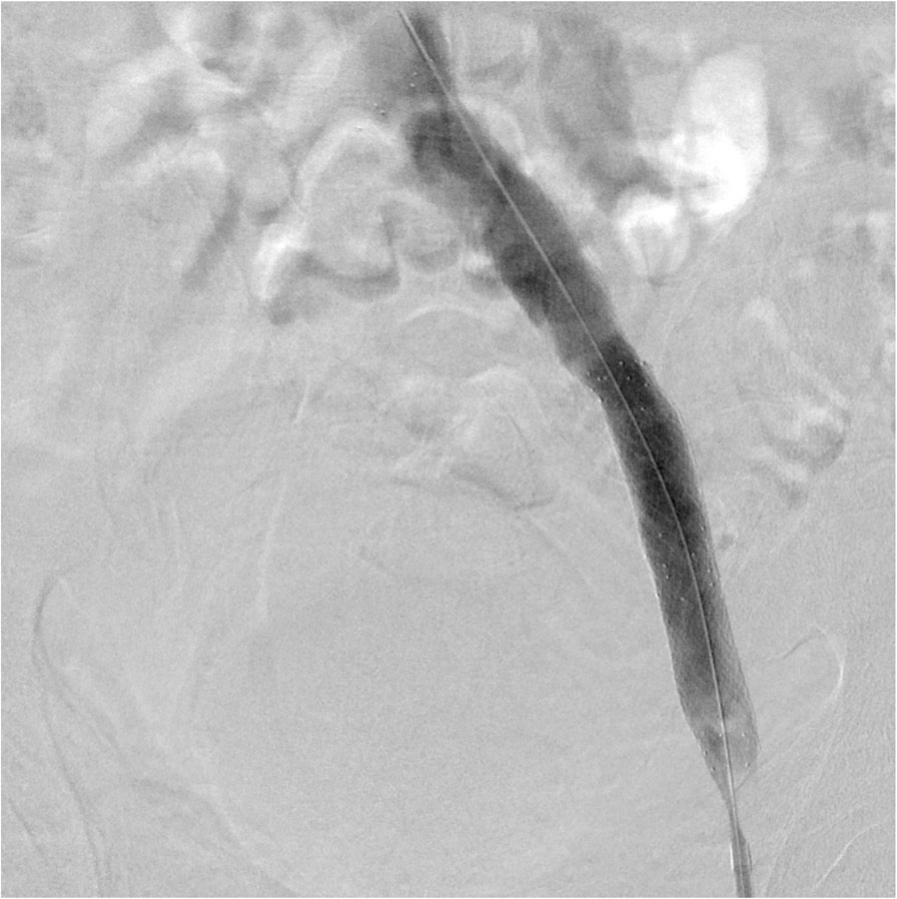
Post-stenting venography. Brisk flow is demonstrated through the stented occlusion. Drainage through the sapheno-saphenous collaterals is no longer observed

After exchanging for a Magic Torque wire (Boston Scientific, Maple Grove, MN), 6 × 40 mm balloon angioplasty (Mustang, Boston Scientific, Maple Grove, MN) of the left common and external iliac veins was performed. The location of the extrinsic left iliac vein compression was identified by simultaneous venography using a reverse curve catheter (5Fr Sos, AngioDynamics, Latham, NY) with its end hole in the right iliac vein and through the ipsilateral access sheath. Subsequently, 14 mm self-expanding stents (SMART Control, Cordis, Fremont, CA) were deployed from the ostium of the left common iliac vein and the external iliac vein, proximal to the inguinal ligament, and were post dilated with a 14 × 40 mm balloon (Atlas, Bard Peripheral Vascular, Tempe, AZ). Completion venography demonstrated brisk flow through the recanalized segment and cessation of flow across the sapheno-saphenous collateral (Fig. 3). CT venography performed at one month demonstrated stent patency; the patient’s edema improved and pain resolved. The post-operative VCSS for the patient was 2, indicating that the procedure enhanced the patient’s quality of life. After the procedure the patient began a daily anticoagulation regimen of aspirin 81 mg and clopidogrel 75 mg indefinitely.

The second patient, a 55-year-old female, with a history of left lower extremity DVT, presented with persistent left lower extremity swelling, pain and lipodermatosclerosis despite conservative management with warfarin. The pre-operative VCSS for the patient was 9, providing indication for intervention. The patient had a history of venous thromboembolic disease status post placement of a permanent, Greenfield type filter approximately 25 years prior; this was retrieved prior to her recanalization procedure.

**Fig. 4:**
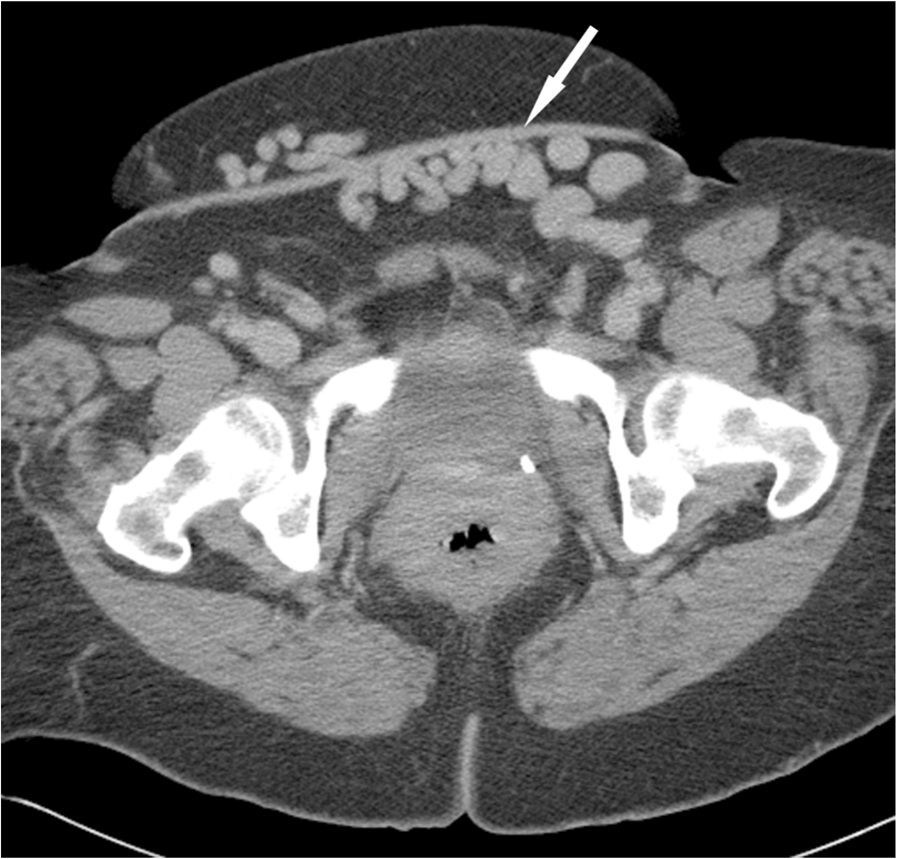
Axial CT of a 55-year old woman presenting with bilateral lower extremity edema and pain. Torturous collaterals connecting the great saphenous veins are noted coursing through the anterior pelvic wall adipose tissue (white arrow)

Prior to the procedure, lower extremity duplex imaging demonstrated partial non-compressibility of the left common femoral vein. CT venography revealed chronic occlusion of the left iliac vein with cross pelvic collaterals draining into the right common femoral vein (Fig. 4).

**Fig. 5:**
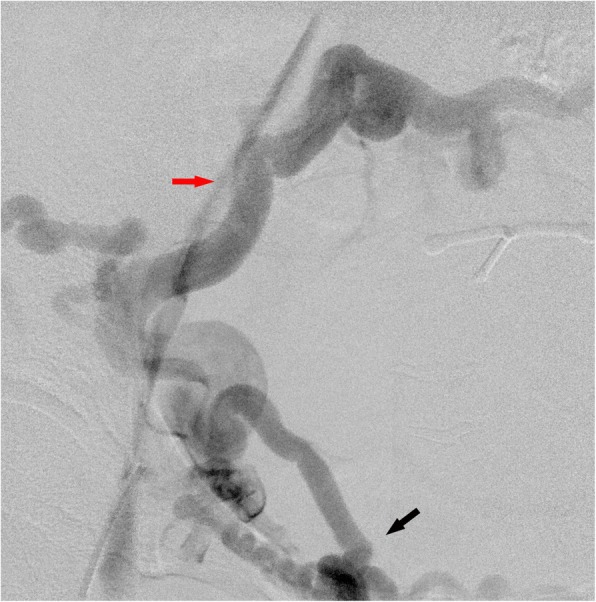
Digital subtraction venography demonstrates near total occlusion of left iliac vein. Near total occlusion of the left iliac vein (red arrow). Dominant drainage is observed through a left-to-right sapheno-saphenous collaterals (black arrow)

During the procedure, access was obtained in the left popliteal vein under sonographic guidance. Venography demonstrated subtotal occlusion (95%) of the left common femoral vein and complete occlusion of the left iliac vein. A large left to right sapheno-saphenous bypass collateral was identified, along with retroperitoneal and pelvic collaterals (Fig. 5).

After placement of an 8Fr sheath, a 5Fr 90-cm Ansel 1 and CXi catheter (Cook Medical, Bloomington, IN) along with a stiff Glidewire were used for traversal of the iliac occlusion. A 10 × 80 mm balloon (Mustang, Boston Scientific, Maple Grove, MN) was used to perform balloon angioplasty of the occluded iliac segment. This was followed by introduction of a reverse curve catheter introduced alongside the wire, which was utilized to identify the position of the great venous confluence. Fourteen mm self-expanding stents were deployed through the iliac segment, and a 12 × 80 mm self-expanding stent (SMART Control, Cordis, Fremont, CA) was then deployed to the level of the lesser trochanter, proximal to the deep femoral venous inflow.

**Fig. 6:**
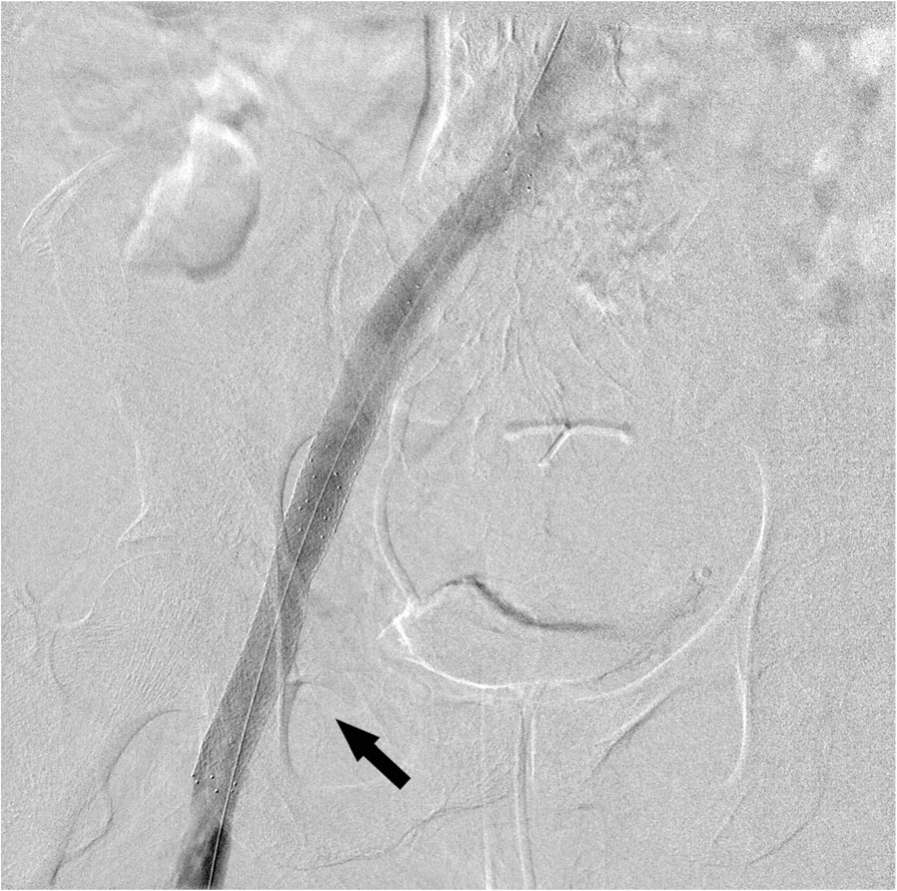
Post stent venography. Brisky flow is demonstrated through the stented iliac vein (black arrow). Drainage through lumbar and sapheno-saphenous collaterals are no longer seen

The stents were post-dilated with 14 and 12 × 40 mm balloons (Atlas, Bard Peripheral Vascular, Tempe, AZ), respectively, and completion venography was performed, demonstrating brisk flow through the recanalized venous segments. Flow through the sapheno-saphenous collateral was no longer visualized (Fig. 6). One month CT venography demonstrated stent patency; the patient’s edema improved and pain resolved. A post-procedure VCSS of 4 demonstrates improvement in the patient’s symptom severity compared to before the intervention. The patient’s post-procedure anticoagulation consisted of warfarin 5 mg daily for an indefinite period.

## Discussion

In the setting of chronic venous occlusion, collateral veins develop as a pathway of lower resistance to maintain circulation. In this situation it serves as a confirmatory indicator of the presence of an occlusion to in-line venous drainage. Whereas lumbar and cross-pelvic internal iliac drainage pathways are well known (Ahmed and Hagspiel 2001; Butros et al. 2013; Englund 2017; Hartung et al. 2009; O’Dea and Schainfeld 2014; Neglen and Raju 2002; Neglén and Raju 2000), to our knowledge, there are no reports of autologous development of a sapheno-saphenous collateral bypass pathway secondary to the venous obstruction.

In a study that assessed chronic iliac vein obstruction, collateral formation was identified in 71% of the limbs (122 of 173), 89% of which were transpelvic collaterals (Neglen and Raju 2002). Another study using intraoperative venogram showed venous collateralization in 62% (63 of 102) of treated limbs, 66% of which were transpelvic collaterals (Neglén and Raju 2000). Sapheno-saphenous collaterals may represent a type of cross-pelvic collateral in a similar physiologic continuum as cross-pelvic internal iliac collateral drainage pathways.

A large case series of 295 patients with collateral veins on the abdominal wall or pubic bone has demonstrated the benefit of collateral vein findings on identification of obstructive venous pathology (Kurstjens et al. 2016). The positive predictive value of an abdominal wall collateral for deep venous obstructive disease at the level of the groin or more proximal was 93% (95% CI, 90–96). These collateral lesions were left untreated in fear of obliterating venous drainage pathways. The case series presented herein re-illustrates the usefulness of collateral vein findings on cross-sectional imaging and also demonstrates that endo-venous intervention with stent placement can ameliorate clinical symptoms effectively while maintaining collateral venous inflow.

## Conclusion

Increases in use of medical imaging, particularly CT, has led to increased recognition of various vascular anomalies. Previously described venous collateral pathways may be difficult to visualize, given their location in the retroperitoneum or close association with pelvic viscera. A diminutive/atretic iliac vein may also be underrecognized, particularly in individuals with a lesser amount of retroperitoneal fat. Identification of a sapheno-saphenous collateral can provide an important clue to the underlying venous obstructive pathology, therefore guiding corrective intervention.

With the growing interest in endovascular treatment of deep venous disease, recognition of secondary signs of venous obstruction, particularly on cross sectional imaging, will become increasingly important. Knowledge of a sapheno-saphenous bypass collateral pathway associated with unilateral iliac vein occlusion is important for diagnostic and interventional radiologists alike and can help guide triage and management of these patients.

## Abbreviations

CT: Computed tomography
DVT: Deep venous thrombosis
